# Modulation of TGFbeta 2 levels by lamin A in U2-OS osteoblast-like cells: understanding the osteolytic process triggered by altered lamins

**DOI:** 10.18632/oncotarget.3232

**Published:** 2015-03-16

**Authors:** Camilla Evangelisti, Pia Bernasconi, Paola Cavalcante, Cristina Cappelletti, Maria Rosaria D'Apice, Paolo Sbraccia, Giuseppe Novelli, Sabino Prencipe, Silvia Lemma, Nicola Baldini, Sofia Avnet, Stefano Squarzoni, Alberto M. Martelli, Giovanna Lattanzi

**Affiliations:** ^1^ Rizzoli Orthopedic Institute, Laboratory of Musculoskeletal Cell Biology, CNR Institute for Molecular Genetics, Unit of Bologna, Bologna, Italy; ^2^ Neurology IV Unit - Neuroimmunology and Neuromuscular Disorders, Foundation IRCCS Neurological Institute “Carlo Besta”, Milan, Italy; ^3^ U.O.C. Medical Genetics Laboratory, AOU Policlinico Tor Vergata, Rome, Italy; ^4^ Department of Internal Medicine, University of Rome Tor Vergata, Rome, Italy; ^5^ Department of Biomedicine and Prevention, University of Rome Tor Vergata, Rome, Italy; ^6^ Rizzoli Orthopedic Institute, Laboratory for Pathophysiology, Bologna, Italy; ^7^ Department of Biomedical and Neuromotor Sciences, University of Bologna, Bologna, Italy

**Keywords:** TGFbeta2, lamin A, osteoclasts, Akt signaling, RAD001

## Abstract

Transforming growth factor beta (TGFbeta) plays an essential role in bone homeostasis and deregulation of TGFbeta occurs in bone pathologies. Patients affected by Mandibuloacral Dysplasia (MADA), a progeroid disease linked to *LMNA* mutations, suffer from an osteolytic process. Our previous work showed that MADA osteoblasts secrete excess amount of TGFbeta 2, which in turn elicits differentiation of human blood precursors into osteoclasts. Here, we sought to determine how altered lamin A affects TGFbeta signaling. Our results show that wild-type lamin A negatively modulates TGFbeta 2 levels in osteoblast-like U2-OS cells, while the R527H mutated prelamin A as well as farnesylated prelamin A do not, ultimately leading to increased secretion of TGFbeta 2. TGFbeta 2 in turn, triggers the Akt/mTOR pathway and upregulates osteoprotegerin and cathepsin K. TGFbeta 2 neutralization rescues Akt/mTOR activation and the downstream transcriptional effects, an effect also obtained by statins or RAD001 treatment. Our results unravel an unexpected role of lamin A in TGFbeta 2 regulation and indicate rapamycin analogs and neutralizing antibodies to TGFbeta 2 as new potential therapeutic tools for MADA.

## INTRODUCTION

The nuclear envelope is a master regulator of several cellular functions thanks to its ability to modulate transcription and chromatin arrangement in response to external stimuli [1]. An interplay with the nuclear envelope has been demonstrated for diverse signaling effectors such as extracellular signal-regulated kinases (ERK) 1/2, c-Fos [2], Wnt/b-catenin [3], Akt/mammalian target of rapamycin (mTOR) [4, 5], TGFbeta 1 and 2 [6–8]. Regarding cytokine regulation by nuclear envelope/lamina proteins, an increasing number of studies in human and mouse models of laminopathies highlight dysregulation of cytokine levels downstream of lamin mutations [9–14]. TGF beta signaling may regulate bone remodeling, acting on osteoblast and osteoclast homeostasis [15]. TGF beta is produced by osteoblasts [16], synthesized in a biologically inactive form [17] and deposited into the bone matrix [18], where it is released and activated during bone resorption [19]. Moreover, TGFbeta 2 is considered a primer of hematopoietic precursor fate, acting on monocytes to commit them to osteoclastogenesis [15].

In this study, we followed our previously published results showing that altered TGFbeta 2 secretion from Mandibuloacral Dysplasia (MADA, OMIM # 248370) osteoblasts induces osteoclastogenesis and bone resorption [11]. MADA is a rare laminopathy associated with postnatal growth retardation, anomalous skin pigmentation and fat distribution and metabolic abnormalities [20, 21]. The MADA phenotype is further characterized by severe bone defects, including generalized osteoporosis and osteolysis at clavicles, phalanges and mandibula [20]. Most MADA cases are linked to the homozygote R527H *LMNA* mutation [22]. Remarkably, MADA and all the other laminopathies featuring bone resorption [6] are characterized, at the molecular level, by accumulation of anomalous levels of the lamin A precursor protein known as prelamin A [23–28]. We previously reported that accumulation of prelamin A during differentiation of peripheral blood monocytes favors osteoclastogenesis and increases secretion of cathepsin K, a protease involved in extracellular matrix resorption [29]. Moreover, in MADA patient's osteoblasts, we reported increased levels of osteoprotegerin (OPG), a TNF receptor superfamily member acting as a decoy receptor regulating osteoclast differentiation. This condition was associated with elevated TGFbeta 2 levels and resulted in imbalanced TGF beta 2-mediated non-canonical osteoclastogenesis and increased resorption activity [11]. To go in deep into this unexpected effect, we introduced the R527H *LMNA* mutation causing MADA [20] into an osteoblast-like cell line, the human U2-OS osteosarcoma cells, and investigated the downstream signaling pathways. To discriminate between the effects related to the R527H *LMNA* mutation per se and those depending on the accumulation of prelamin A, we further expressed wild-type lamin A or an unprocessable prelamin A mutant in U2-OS cells and followed their effect. Unexpectedly, we found that lamin A down-regulates TGFbeta 2 levels in cells and secretion in medium, while its R527H mutant found in MADA fails to modulate TGFbeta 2 causing increased secretion of this cytokine, in accordance with our previous results [11]. Our data demonstrate that TGFbeta 2 increase in R527H cells is associated with activation of the Akt/mTOR pathway and this effect is reversed by TGFbeta 2 neutralizing antibody, statins and the mTOR inhibitor RAD001, which also avoid the aberrant osteoclastogenesis triggered by laminopathic culture media.

## RESULTS

Our results show that lamin A is able to modulate TGFbeta 2 levels, while its mutated form found in MADA causes excess levels of this cytokine and triggers elevated OPG and cathepsin K amount through activation of the Akt/mTOR pathway. TGFbeta neutralizing antibody, RAD001 or mevinolin treatment rescues the affected pathway as well as TGFbeta 2-dependent osteoclastogenesis triggered by conditioned media.

### Overexpression of prelamin A affects the secretory profile in human osteoblast-like cells

**Figure 1:**
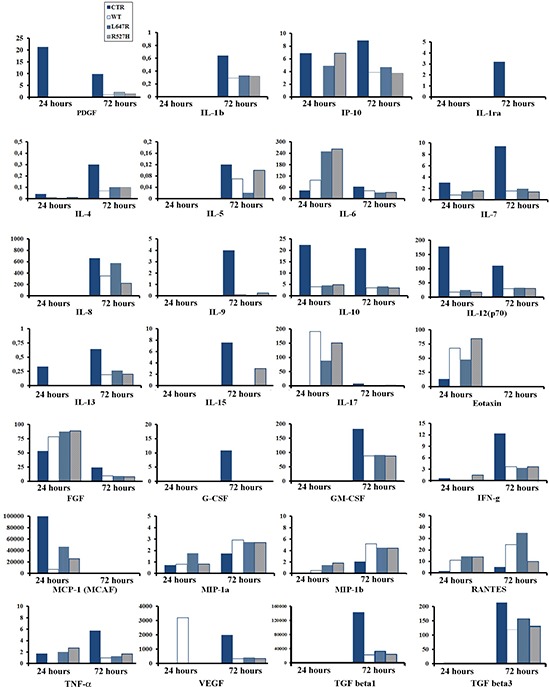
Cytokine secretion in U2-OS osteoblast-like cells is influenced by *LMNA* expression Cell culture media mock-transfected U2-OS (CTR), or U2-OS transfected with WT, L647R or R527H LMNA plasmids were subjected to multiplex cytokine assay. Results for 28 cytokines/growth factors are reported in the graphs. Media were collected 24 or 72 hours after transfection. Protein values indicated on the Y axes are reported as pg/ml. Graphs are representative of three different experiments.

**Figure 2:**
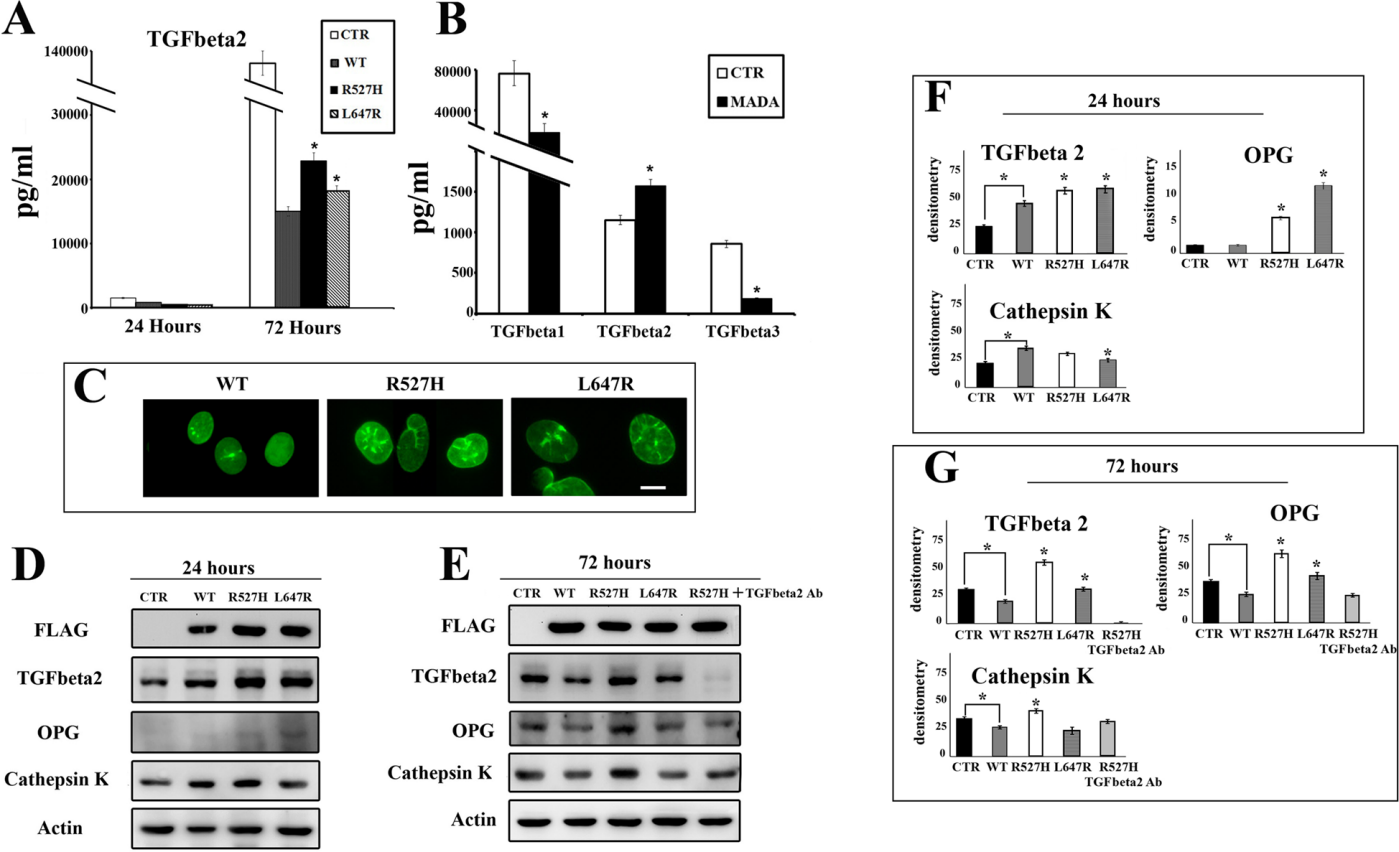
R527H *LMNA* leads to increase of TGFbeta 2, OPG and cathepsin K levels **(A)** Amount of secreted TGFbeta 2 as detected by multiple cytokine assay in media derived from mock-transfected U2-OS cells (CTR) or cells transfected with *LMNA* plasmids (WT, R527H, L647R) (media were collected 24 or 72 hours after transfection). **(B)** TGFbeta 1, 2 and 3 levels determined by multiple cytokine assay in serum from healthy donors (22 subjects) (CTR) or MADA patients (4 subjects) (MADA). **(C)** Immunofluorescence staining of FLAG fusion proteins in WT, R527H or L647R *LMNA* U2-OS cells. Cells were labeled using anti-FLAG antibody revealed by a FITC-conjugated secondary antibody. Bar, 10 μm. **(D–E)** Western blot analysis of TGFbeta 2, OPG and cathepsin K in mock-transfected (CTR) or *LMNA*-transfected U2-OS cells 24 or 72 hours after transfection. FLAG bands show the overexpressed proteins, actin bands show equal sample loading. Protein levels in R527H-expressing cells subjected to TGFbeta 2 neutralization by anti-TGFbeta 2 antibody are shown in E. **(F–G)** Densitometric values of immunoblotted bands showed in D and E. Data are means of three independent experiments performed on different samples +/− standard deviation. Statistically significant values ( *p* < 0.05) were calculated by Dunnet test. Asterisks show significance ( *p* < 0.05) relative to WT *LMNA* sample values, except when differently indicated by connectors.

To get insights into the effect of lamins on TGFbeta 2 regulation, U2-OS cells were transfected with FLAG-tagged plasmids expressing wild-type prelamin A (WT), which is produced as mature lamin A, or uncleavable prelamin A (L647R), which yields accumulation of farnesylated prelamin A. Moreover, to investigate the molecular pathway triggering altered cytokine regulation in MADA osteoblasts, we introduced the R527H *LMNA* mutant in U2-OS. At first, to test the secretory profile of transfected U2-OS cells, we examined conditioned medium from those cell cultures by multiplex cytokine assay (Figure 1). Our data showed a general effect of *LMNA* expression on the secretory profile of osteoblast-like cells and pointed to an inhibitory effect for most cytokines and growth factors including TGFbeta 1 and 3 (Figure 1) and TGFbeta 2 (Figure 2A). Only in the case of Mip-1a and b and RANTES (CCL3) lamin A overexpression elicited chemokine increase (Figure 1), an interesting finding based on the role of these molecules in osteolytic processes [30]. In most cases, overexpression of R527H *LMNA* or farnesylated prelamin A accumulation (L647R *LMNA*) elicited the same effect on cytokine secretion as wild-type lamin A (Figure 1).

However, the R527H *LMNA* mutation, as well as farnesylated prelamin A accumulation, failed to properly regulate TGFbeta 2 levels, causing significantly increased amounts of secreted TGFbeta 2 in U2-OS culture medium (Figure 2A). This finding is in accordance with the reported increase of TGFbeta 2 in MADA osteoblasts [11]. Moreover, serum levels of TGFbeta were altered in a group of MADA patients carrying the R527H LMNA mutation: TGFbeta 1 and 3 levels were reduced, whereas the amount of TGFbeta 2 was increased (Figure 2B).

Then, we investigated the effect of *LMNA* expression on TGFbeta 2 and downstream signaling events in cells. In transfected U2-OS, FLAG-tagged lamins were localized at the nuclear periphery, but also formed aggregates in R527H and L647R cells (Figure 2C), resembling the condition observed in MADA skin fibroblasts [22]. Overexpression of wild-type prelamin A in U2-OS favored TGFbeta 2 production 24 hours after transfection, in the condition of low cytokine synthesis, as detected by western blot analysis (Figure 2D and 2F). However, wild-type lamin A overexpression reduced TGFbeta 2 in cells and secretion in medium 72 hours after transfection (Figure 2A, and 2G). Conversely, L647R prelamin A and mostly the R527H prelamin A mutant failed to down-regulate TGFbeta 2 levels (Figure 2A, and 2G). These results implicated lamin A in the modulation of TGFbeta 2 levels and showed that the *LMNA* mutation causing MADA, as well as farnesylated prelamin A, could affect this function leading to anomalous TGFbeta 2 levels in osteoblast-like cells.

To investigate the relevance of this finding to osteoclastogenesis, we analyzed the expression of two markers of bone turnover, OPG and cathepsin K that are involved in bone remodeling and resorption [11, 31]. As for TGFbeta 2, WT lamin A exerted a positive effect on cathepsin K production at the early time point and a negative effect on both cathepsin K and OPG production at 72 hours after transfection (Figure 2D–2G).

However, overexpression of R527H-mutated *LMNA* induced a striking OPG increase (Figure 2D–2G). Moreover, cathepsin K increase was evident in R527H cells 72 hours after transfection (Figure 2E and 2G). The L647R *LMNA* mutation exerted a milder effect on TGFbeta 2, OPG and cathepsin K levels (Figure 2D–2G). These results indicated the R527H *LMNA* mutation per se rather than prelamin A accumulation causes OPG and cathepsin K increase in MADA.

To confirm the role of TGFbeta 2, we inhibited its activity in R527H cells by using a neutralizing antibody. Remarkably, in TGFbeta 2-neutralized R527H U2-OS cells, OPG and cathepsin K levels were comparable to those measured in WT cells, showing that OPG and cathepsin K regulation by lamin A occurred through TGFbeta 2 signaling (Figure 2E and 2G).

### The Akt/mTOR pathway mediates TGFbeta 2 effects in R527H U2-OS cells

**Figure 3:**
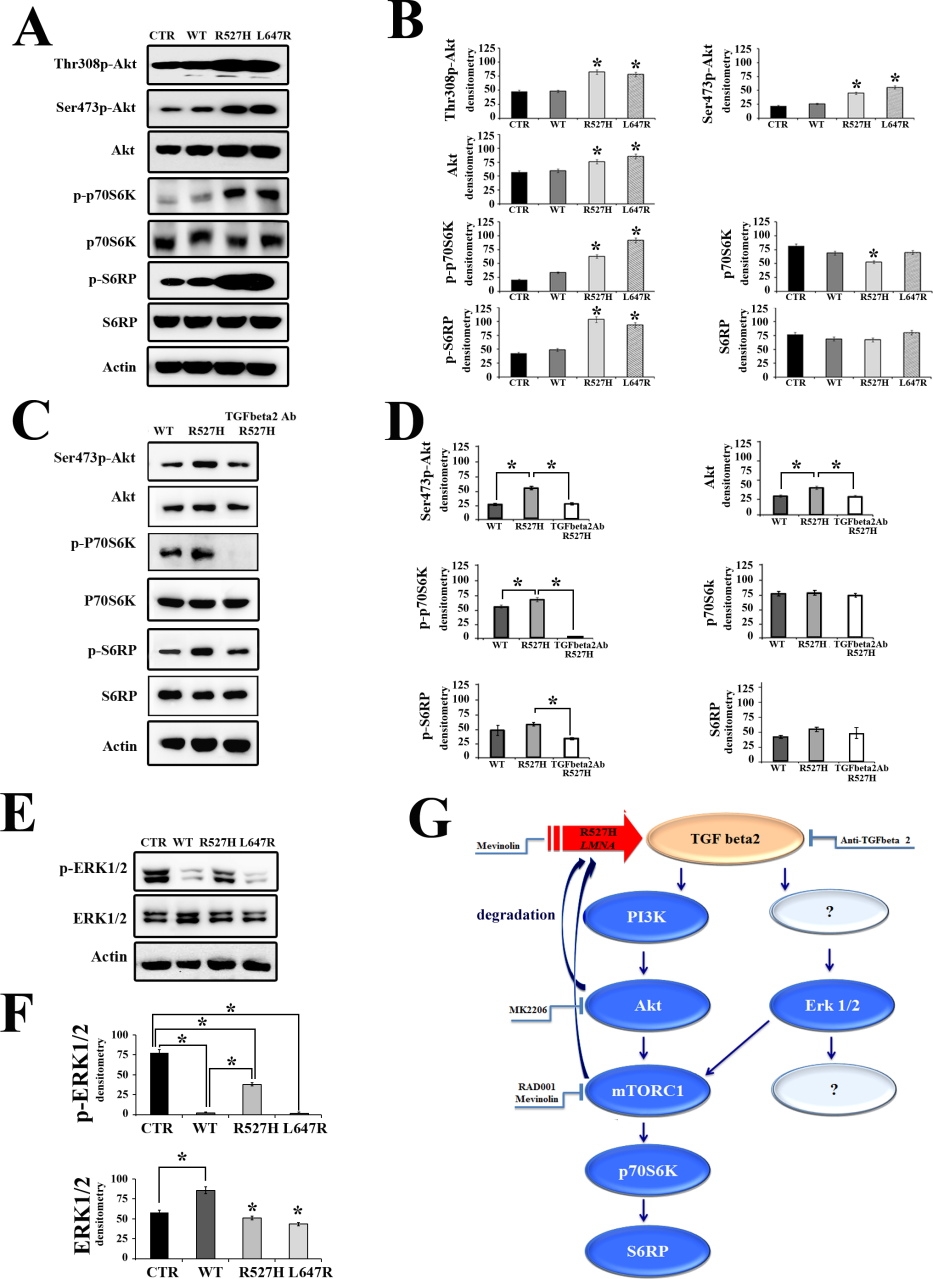
The Akt/mTOR pathway is modulated by lamins through TGFbeta 2 **(A)** Western blot analysis of U2-OS cells transfected with mock or WT, R527H or L647 *LMNA* plasmids showing the amount of active Akt (Thr308p-Akt and Ser473p-Akt), p70S6K (p-p70S6K) and S6RP (p-S6RP) and their total amount (Akt, p70S6K and S6RP, respectively). Actin bands show equal loading. The corresponding densitometric analysis of immunoblotted bands is reported in **(B)** as mean values of three different experiments +/– standard deviation. **(C)** Neutralization of TGFbeta 2 activity in R527H U2-OS cells blocks the activation of the Akt/mTOR pathway. U2-OS expressing WT or R527H *LMNA* were subjected to western blot analysis. R527H U2-OS were left untreated (R527H) or treated with anti-TGFbeta 2 neutralizing antibody (right lane, TGFbeta2 Ab R527H) as detailed in the methods section. The corresponding densitometric analysis of immunoblotted bands is reported in **(D)** as mean values of three different experiments +/– standard deviation. **(E)** Phosphorylated ERK 1/2 (p-ERK 1/2) and ERK 1/2 levels in U2-OS 24 hours after transfection of mock (CTR), WT, R527H or L647R *LMNA* plasmids. ERK1/2 activity is reduced by expression of WT *LMNA*, but not by R527 *LMNA*. Statistically significant differences (*P* < 0.05) are indicated by asterisks in (in B, D, **(F)**). **(G)** Schematic representation of the TGFbeta 2-dependent signaling pathway affected by R527H-mutated *LMNA* expression and possible targets of drug intervention. R527H-mutated *LMNA* fails to regulate TGFbeta 2 levels causing elevated levels of TGFbeta 2 in U2-OS cells. Downstream events are activation of the Akt pathway and ERK 1/2 activation, which influence mTORC1 activity. The Akt/mTOR pathway can be blocked at diverse levels using MK2206 to inhibit Akt, RAD001 or statins (mevinolin) to inhibit mTORC1. Mevinolin also acts on mutated prelamin A avoiding protein farnesylation and favoring phosphorylation by Akt and lysosomal degradation of prelamin A (4, 5). RAD001 is also expected to trigger prelamin A degradation (33). Anti-TGFbeta 2 neutralizing antibody can be used to block the whole signaling pathway.

Several signaling effectors act downstream of TGFbeta receptor activation [32]. Here, we could demonstrate involvement of the Akt/mTOR pathway in TGFbeta 2-dependent OPG and cathepsin K increase by testing the effectors of that pathway in R527H U2-OS. The examined signaling molecules, including Akt, P70S6 kinase (P70S6K) and S6 ribosomal protein (S6RP) were not affected by expression of WT *LMNA*, but were significantly activated in R527H-*LMNA* transfected cells and in cells expressing farnesylated prelamin A (Figure 3A–3D). Neutralization of TGFbeta 2 activity inhibited the Akt/mTOR pathway in R527H *LMNA* U2-OS (Figure 3A–3D). Moreover, we observed a striking reduction of ERK 1/2 phosphorylation in cells overexpressing WT *LMNA* and unprocessable prelamin A, but not in R527H U2-OS (Figure 3E–3F). Activation of the MAPkinase-ERK 1/2 pathway is an effect previously described in mouse models of muscular laminopathies (13). Intriguingly, ERK 1/2 activity may converge on mTOR as does the Akt pathway (Figure 3G). These findings and other considerations reported below prompted us to block the Akt/mTOR pathway using several inhibitors (Figure 3G) and test the effect on OPG and cathepsin K modulation.

### RAD001 treatment rescues TGFbeta 2, OPG and cathepsin K in R527H mutated U2-OS cells

**Figure 4:**
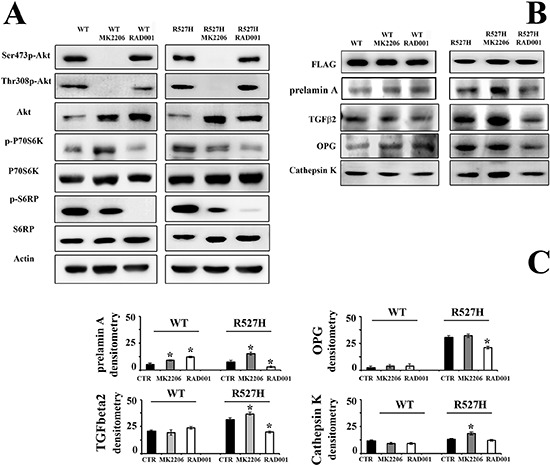
RAD001 treatment of R527H *LMNA* rescues the Akt/mTOR signaling pathway, and OPG levels and reduces cathepsin K amount **(A)** Western blot analysis of the Akt/mTOR signaling pathway effectors in U2-OS cells transfected with WT *LMNA* (left panels) or R527H *LMNA* (right panels). Cells were subjected to Akt inhibitor MK2206 or mTORC1 inhibitor RAD001. The immunoblotted bands correspond to active Akt (Thr308p-Akt and Ser473p-Akt), total Akt (Akt), active p70S6K (p-p70S6K) and active S6RP (p-S6RP) and total p70S6K and S6RP. Actin bands show equal loading. **(B)** Western blot analysis of prelamin A, TGFbeta 2, OPG and cathepsin K in the samples analyzed in (A) The densitometric analysis of immunoblotted samples shown in (B) is reported in **(C)** as mean values of three different experiments +/– standard deviation. Statistically significant values relative to each untreated sample (CTR), are indicated by asterisks.

To support the above reported involvement of Akt/mTOR pathway in the altered regulation of OPG and cathepsin K, we inhibited Akt activity, using MK2206 and mTOR, using the rapamycin analog RAD001 that impairs mTORC1 activity (Figure 4). While Akt activity was completely inhibited by MK2206, protein amount was increased (Figure 4A). Akt inhibition caused dysregulation of its downstream effectors, including P70S6K and S6RP, although with different effects in WT lamin A versus R527H-mutated lamin A-transfected cells (Figure 4A). Phosphorylation of P70S6K was increased in WT-transfected cells and decreased in R527H-transfected U2-OS, while S6RP phosphorylation was consistently inhibited in all MK2206-treated cells (Figure 4A). RAD001 minimally affected Akt activity, but elicited protein increase (Figure 4A), possibly due to a feedback mechanism. Complete inhibition of P70S6K and S6RP phosphorylation was observed in all RAD001-treated cells (Figure 4A).

In R527H-mutated cells, the Akt inhibitor MK2206 led to prelamin A accumulation (Figure 4B–4C). Moreover, TGFbeta 2, OPG and cathepsin K levels were increased (Figure 4B–4C), showing that Akt inhibition intensifies the negative effect of R527H *LMNA* mutation, possibly due to the impairment of Akt-triggered phosphorylation of prelamin A required for protein degradation [5, 33]. Conversely, RAD001 treatment of R527H lamin A-transfected U2-OS rescued prelamin A, TGFbeta 2, OPG and cathepsin K up to levels comparable to WT *LMNA* cells (Figure 4B–4C).

### Statin treatment rescues TGFbeta 2, OPG and cathepsin K in R527H-mutated U2-OS cells

Current clinical trials in laminopathic patients have indicated a beneficial effect of statins associated with their ability to counteract accumulation of farnesylated prelamin A [34].

Based on these considerations and on the observed effect of farnesylated L647R prelamin A on TGFbeta 2 increase, we wanted to test the effect of statins in our cellular model of MADA.

**Figure 5:**
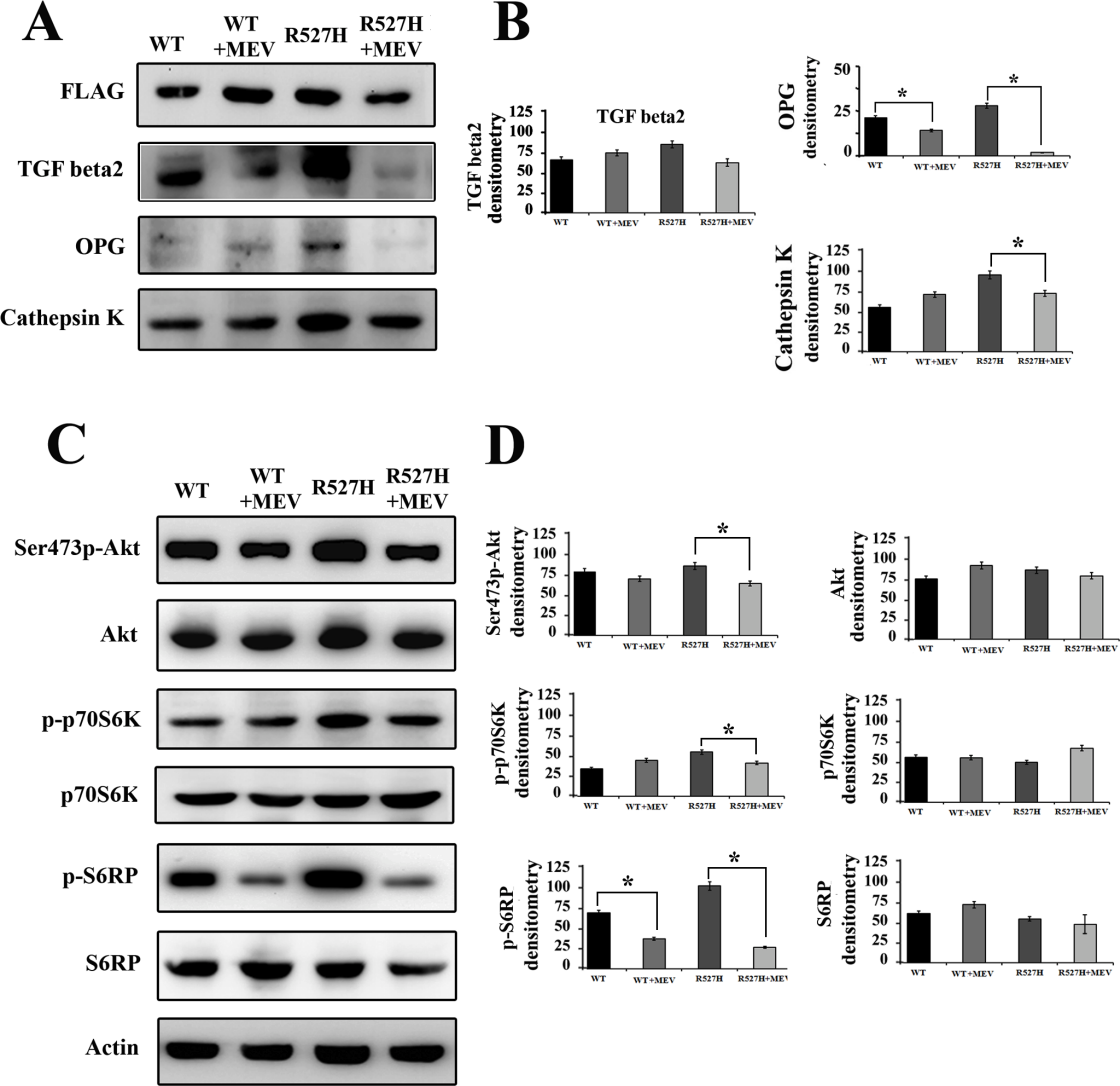
Mevinolin treatment reduces TGFbeta 2 levels and attenuates Akt/mTOR signaling, OPG and cathepsin K levels in R527H LMNA U2-OS cells. U2-OS cells transfected with WT or R527H *LMNA* were left untreated or subjected to 24 hours mevinolin treatment (+MEV) **(A)** Western blot analysis of FLAG-lamin A, TGFbeta 2, OPG and cathepsin K **(B)** Densitometric analysis of immunoblotted proteins shown in (A). **(C)** Western blot analysis of Akt (Thr308p-Akt and Ser473p-Akt), total Akt (Akt), active p70S6K (p-p70S6K) and active S6RP (p-S6RP) and total p70S6K and S6RP. Actin bands show equal loading in (A) and (B). **(D)** Densitometric analysis of immunoblotted proteins shown in (C). Values in (B) and (D) are means +/− standard deviation of three values reported in different experiments. Statistically significant differences ( *p* < 0.05) relative to each untreated sample (WT or R527H), are indicated by asterisks.

U2-OS cells were transfected with WT- and R527H-*LMNA* plasmids and treated with mevinolin for 24 hours. Western blot analysis showed that in mevinolin-treated R527H U2-OS, TGFbeta 2, OPG and cathepsin K levels were significantly lowered (Figure 5A–5B).

Moreover, in agreement with the reported inhibitory effect of statins on mTOR [35], while Akt activity was slightly affected in mevinolin-treated cells, the mTOR effectors P70S6K and S6RP where strikingly inhibited by drug treatment (Figure 5C–5D).

This data indicated that inhibition of prelamin A farnesylation and mTOR activity elicited by statins could partially rescue the aberrant signaling observed in cells expressing R527H prelamin A and downstream effects on OPG and cathepsin K.

### Osteoclastogenic potential of R527H U2-OS medium is reduced by drug treatment

**Figure 6:**
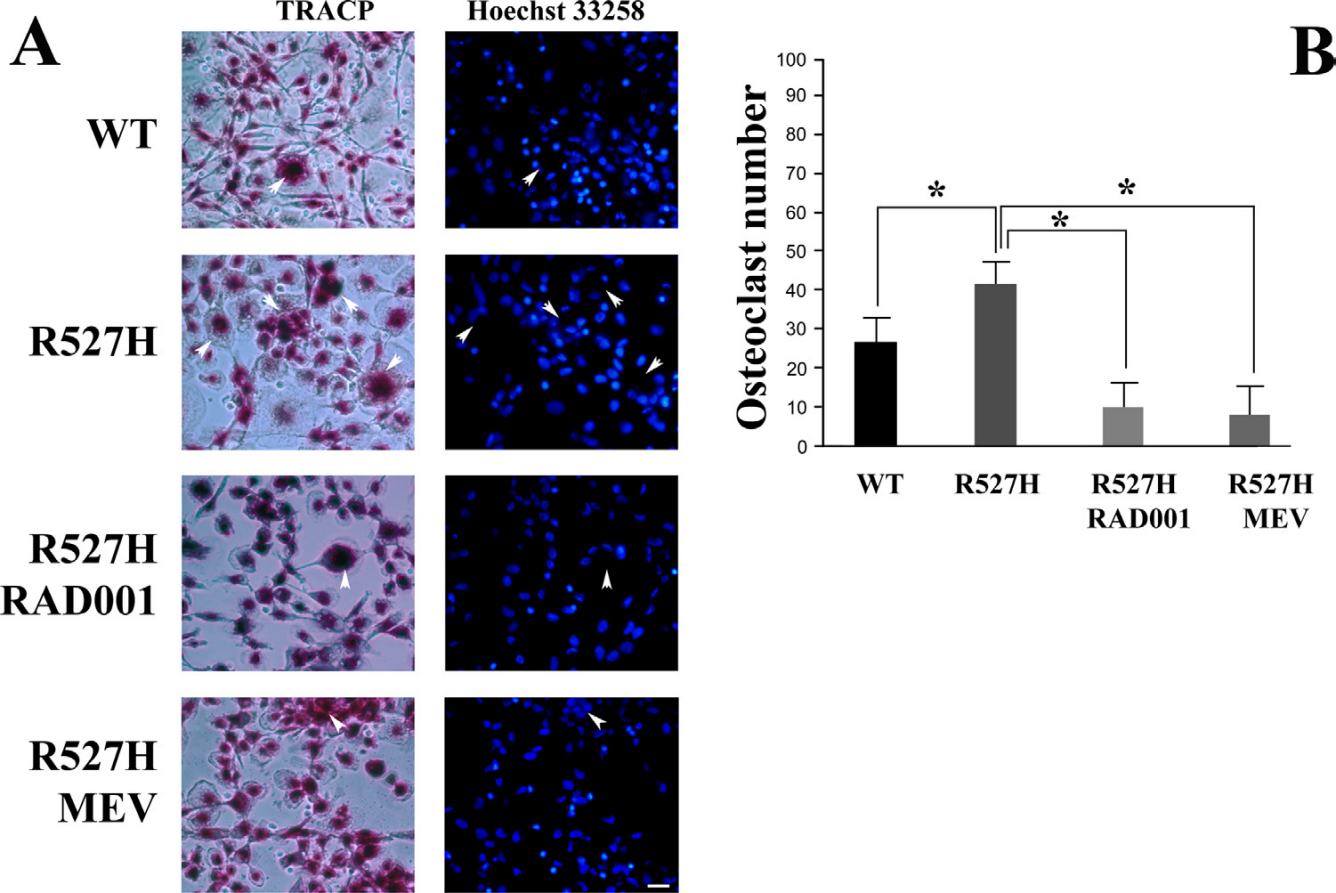
Reduced number of TRACP-positive multinuclear cells (osteoclasts) in human peripheral blood monocyte cultures subjected to conditioned medium from RAD001- or mevinolin-treated R527H *LMNA* U2-OS cells **(A)** Human mononuclear cells were induced to differentiate into osteoclasts for 8 days by adding medium from untreated WT *LMNA*, R527H *LMNA* U2-OS, or R527H *LMNA* U2-OS treated with RAD001 (72 hours) or mevinolin (24 hours). TRACP expression is revealed by red precipitates in representative pictures, nuclei are counterstained with Hoechst 33258 (Bar, 20 μm). Arrowheads indicate osteoclasts and their nuclei. **(B)** Quantitative analysis of osteoclast formation. Osteoclast number per sample is reported on the Y axis. Analysis was performed on six countings of the TRACP positive cells that showed three or more than three nuclei. Data are expressed as mean+/− standard deviation. Statistically significant differences (*p* < 0.05) are indicated by asterisks.

To test the efficacy of RAD001 or mevinolin treatment in terms of inhibition of osteoclastogenesis, we used conditioned media from R527H U2-OS cells to trigger osteoclast differentiation in peripheral blood monocytes from healthy donors. As shown in Figure 6, R527H U2-OS-conditioned precursors differentiated more efficiently than WT U2-OS-conditioned monocytes. However, using conditioned medium from RAD001-treated R527H cells, osteoclast differentiation was significantly reduced (Figure 6A and 6B).

Moreover, medium from mevinolin-treated R527H U2-OS was less efficient in osteoclast induction (Figure 6A and 6B). These results were in agreement with the observed rescue of Akt/mTOR signaling elicited by RAD001 or mevinolin treatment. It should be noted that both RAD001 and mevinolin had been removed from medium after U2-OS treatment, so that we can rule out direct drug effects on osteoclast differentiation.

## DISCUSSION

Our results provide evidence that lamin A modulates TGFbeta 2 levels in human osteoblast-like cells and that this regulatory mechanism is disrupted by mutated lamin A found in MADA or excess levels of farnesylated prelamin A.

We further show that R527H mutated lamin A causes upregulation of TGFbeta 2-dependent Akt/mTOR signaling and affects ERK1/2 phosphorylation.

Downstream of these affected pathways, increased levels of OPG and cathepsin K are produced in human osteoblast-like cells.

These data are in agreement with our previous findings showing elevated TGFbeta 2-dependent osteoclastogenic potential of MADA osteoblasts and increased differentiation of osteoclasts accumulating farnesylated prelamin A [11, 29]. Here, we further demonstrate that secreted TGFbeta 2-triggered osteoclastogenic differentiation can be counteracted by mevinolin or RAD001, both drugs affecting the Akt/mTOR pathway as well as farnesylated prelamin A levels.

In the reported study, we unravel an unexpected role of lamin A in the modulation of TGFbeta 2 levels and, more generally, of the whole secretory profile of osteoblast-like cells. In fact, *LMNA* overexpression exerted a negative effect on TGFbeta 2 levels, as also observed for most the examined cytokines, while triggering positive effects in a few cases, such as RANTES or MIP-1.

The increase in TGFbeta 2 levels observed in patient serum reinforces the relevance of the reported data for the understanding of the bone phenotype in MADA. However, we must take into account that *LMNA* mutations lead to a more extended deregulation of the serum cytokine profile, as shown for TGFbeta 1 and 3, which are negatively affected in MADA, as well as of local cytokine secretion in cells. Thus, this study represents a first step towards the understanding of the systemic effects elicited by *LMNA* mutations. TGFbeta 2 regulation by lamin A could occur through transcriptional mechanisms or activation of microRNAs [36] and deserves further investigation.

Three distinct forms of TGF (TGFbeta 1, 2 and 3) can bind to the heterodimeric TGFbeta receptor complex, consisting of the type I and type II receptor. Once TGFbetas bind to the receptor complex, intracellular signals can be transduced through Smads or other Smad-independent pathways such as Akt/mTOR, RhoA, TAK1, and Ras/MAPK [15, 32]. Here, we observed activation of the Akt/mTOR pathway downstream of TGFbeta 2. Prelamin A phosphorylation on serine 404 by Akt has been implicated in lysosomal degradation of the lamin A precursor [5, 33, 37]. Moreover, Akt has been shown to regulate *LMNA* transcription [5]. Thus, we can hypothesize that mutated lamins or excess levels of farnesylated prelamin A may trigger TGFbeta 2 increase in order to reduce their own levels below the threshold of toxicity. Along this line, inhibiting Akt activity worsened the observed cellular phenotype including altered TGFbeta 2 secretion. However, inhibiting mTOR activity by RAD001 elicited improvement of the secretory phenotype, as did neutralization of TGFbeta 2 by a specific antibody. The positive effect elicited by RAD001 can be considered a good rescue mechanism avoiding the deleterious effects of Akt inhibition. On the other hand, the efficacy of neutralizing anti-TGFbeta 2 antibody could be due to the mild inhibition exerted on Akt by this treatment, associated with rescue of the downstream signaling effectors such as P70S6K and S6RP. Moreover, also the positive outcome of mevinolin treatment can be linked to its inhibitory effect on mTOR activity [38], besides its known inhibitory effect on prelamin A farnesylation [39].

We further show that wild type lamin A and farnesylated prelamin A are able to down-regulate ERK 1/2 phosphorylation, a function lost in MADA mutants. Altered modulation of ERK signaling by mutated lamin A has been reported in cellular and mouse models of muscle laminopathies [8, 13, 40, 41] and it has been linked to the role of lamins as platforms for cFos and ERK 1/2 [2]. The study of the interaction between lamin A and cFos or ERK in MADA cells will provide further insights on the altered ERK activity. Of note, both ERK and Akt contribute to mTOR hyperactivation, thus identifying mTOR as a potential common target of drug intervention, as supported by the beneficial effects of RAD001.

Downstream of Akt/mTOR signaling, we report increased expression levels of cathepsin K. TGF beta-induced cathepsin K secretion has been recently reported in human osteosarcoma cells [42]. Cathepsin K, which is a collagen I and elastin proteolytic enzyme, can be also produced by osteoblasts [43] and its overexpression contributes to extracellular matrix remodeling and osteoblast differentiation [44]. Thus, the relevance of cathepsin K overexpression in MADA could be related not only to its known role in bone resorption, but also to its effects on osteogenesis. Of note, elevated differentiation of MADA osteoblasts [11] and mesenchymal stem cells bearing *LMNA* mutations was previously reported [45]. On the other hand, the occurrence of elevated cathepsin K levels in MADA could cause defects in target tissues other than bone. For instance, altered remodeling of elastin and collagen matrix could occur in MADA fibroblasts due to overexpression of cathepsin K [46] and contribute to the skin and connective tissue phenotype [11, 47].

Also OPG increase here observed in U2-OS cells overexpressing R527H prelamin A or accumulating farnesylated prelamin A is consistent with previous observations in MADA osteoblasts [11] and senescent cells accumulating prelamin A [31]. The role of OPG overexpression in bone turnover is related to its action of decoy receptor for RANKL regulating RANKL-dependent osteoclastogenesis. In MADA osteoblasts, elevated OPG levels shifted osteoclastogenesis towards a TGFbeta 2-dependent mechanism [11]. However, a more complex picture is emerging from recent studies, providing evidence for OPG involvement in several pathogenetic pathways, including vascular calcification [31, 48]. The latter consideration is particularly relevant for Hutchinson-Gilford progeria, a progeroid laminopathy caused by accumulation of progerin, an alternatively spliced farnesylated prelamin A form [28, 31]. Noteworthy, progeria patients are affected by severe and life-threatening vascular disease [49].

TGFbeta 2 osteoclastogenic potential has been demonstrated in transgenic mice showing clavicular hypoplasia and an osteoporosis-like phenotype [50], an animal model recapitulating bone defects of MADA and other progeroid or developmental laminopathies [6]. Levels of TGFbeta influence bone turnover by acting on osteoclast and osteoblast metabolism as well as on hematopoietic osteoclast precursors [11, 15, 43]. In fact, TGFbeta 2 determines commitment of peripheral blood monocytes to RANKL-induced osteoclast formation [15, 51]. Moreover, in our previous work, we showed that TGFbeta 2 is able to increase osteoclast differentiation independently of the RANKL signal, which is blunted by elevated OPG levels in MADA osteoblasts [11]. Consistent with those findings, osteoclastogenic differentiation of mononucleated blood precursors is induced by media from R527H-*LMNA* transfected osteoblast-like cells containing elevated TGFbeta 2 amounts.

### Concluding remarks

The signaling defects reported in this study appear to be mostly related to expression of R527H-mutated *LMNA* causing MADA, although TGFbeta 2 and OPG increase are also triggered by farnesylated prelamin A accumulation. TGFbeta 2-dependent activation of the Akt/mTOR signaling pathway here shown in R527H-*LMNA* cells could be considered a rescue mechanism aimed at reducing the toxic effect of mutated lamins. However, the downstream hyperactivation of the Akt/mTOR pathway elicits deleterious effects including increased secretion of OPG and cathepsin K and elevated osteoclastogenesis. Rescue of pathogenetic pathway and increased osteoclastogenesis by mevinolin may account for the promising outcome of current clinical trials using statins in MADA patients. It has been also reported a beneficial effect of pravastatin on osteoblast differentiation of mesenchymal stem cells accumulating farnesylated prelamin A [52]. However, since systemic treatment with statins also targets osteoclast precursors, whose differentiation is enhanced by accumulation of non-farnesylated prelamin A [29], this approach might be not suitable to completely recover the bone phenotype in MADA. On the other hand, this study supports our recently published data showing that rapamycin treatment can be explored as a therapeutic approach for MADA [33].

The relevance of the reported results for translation into therapeutic strategies is highlighted by the observation that TGFbeta 2 levels are higher in MADA sera than in healthy blood donors' sera. Moreover, based on recent data showing accumulation of prelamin A in old and very old individuals [53], it is conceivable that the reported prelamin A-triggered signaling might be involved even in the osteoporotic process occurring in old subjects. This observation has obvious implications for the investigation of therapeutic strategies. Overall, our data here reported add to existing evidence that nuclear envelope proteins are involved in the regulation of TGFbeta signaling [54, 55].

## MATERIALS AND METHODS

### Patients

Serum samples were collected from previously characterized MADA patients [20] and two newly enrolled patients following informed consent. Italian patients (two males and two females) with different age, ranging from 5 to 40 years, were included in the study. All the patients were homozygous for the R527H *LMNA* mutation and phenotypically showed acro-osteolysis, cranio-facial abnormalities, hypoplasia of clavicles, and type A lipodystrophy. Mild signs of accelerated ageing were also observed. Serum from 22 healthy blood donors (3 males and 19 females, ranging from 23 to 50 years) was also tested. All the local and EU ethical issues were respected in the study and approval for the cytokine screening was obtained from the Carlo Besta Institute Ethical committee (Milan).

### Cell culture, transfection and treatments

The human osteosarcoma U2-OS cell line was grown in Dulbecco's modified Eagle's medium - High Glucose (DMEM-HG), supplemented with 10% fetal bovine serum (FBS) and antibiotics mix at 37°C and 5% CO_2_. Transient transfections were performed according to the protocol from Amaxa, using an Amaxa Nucleofector apparatus. Transfected cells were plated in 12-well tissue culture plates or in flask (80% confluence). Biochemical analyses were performed 24 or 72 hours after transfection while mevinolin (10 μM), an inhibitor of the hydroxymethyl-glutaryl-synthase that inhibits farnesylation of prelamin A, was added for 24 hours. Treatment with MK-2206 (1 μM), an Akt inhibitor, was applied for 24 hours; RAD001 (1 μM) was applied for 72 hours.

### Antibodies and reagents

Mevinolin was from Sigma-Aldrich while MK2206 and RAD001 were purchased from Selleck Chemicals. For western blotting, primary antibodies anti-prelamin A (Sc-6214) and anti-OPG were from Santa Cruz Biotechnologies. Anti-Ser 473 p-Akt, -Thr 308 p-Akt, anti-Akt, anti-p-S6RP, anti-S6RP, anti-FLAG, anti-p-P70S6K, anti-P70S6K, anti-actin, rabbit polyclonal and mouse monoclonal were from Cell Signaling Technologies. Anti-TGFbeta 2 (ab 10850) and anti-cathepsin K antibodies were from Abcam.

### Plasmids

The following plasmids were used to transfect U2-OS cells: WT LMNA, L647R LMNA, encoding a farnesylated, carboxy-methylated unprocessed prelamin A [56] and R527H LMNA, encoding a mutant lamin A form associated with MADA. All the expressed proteins were fused to a FLAG tag [57].

### Western blot analysis

Whole cell lysates were prepared by the addition of RIPA buffer (20 mM Tris-HCl, pH 7.0, 1% Nonidet P-40, 150 mM NaCl, 10% glycerol, 10 mM EDTA, 20 mM sodium fluoride, 5 mM sodium pyrophosphate, 1 mM Na3VO4, 1 mM PMSF, 10 μg/ml leupeptin and 10 μg/ml pepstatin) at 4°C for 30 minutes. Protein concentration of samples was determined using the Bradford protein assay (Bio-Rad).

Fifty micrograms of total proteins were diluted in sample buffer, subjected to SDS-PAGE and transferred to nitrocellulose membrane. Membranes were saturated with 4% BSA and incubated with primary antibodies overnight at 4°C. Secondary antibodies were incubated for 1 hour at room temperature. Immunoblotted bands were revealed by the Amersham ECL detection system. Analysis with an antibody to actin demonstrated equal protein loading. Intensity measurement was performed using a BioRad densitometer (GS 800) equipped with Quantity One Software.

### Immunofluorescence

Transfected U2-OS cells grown on coverslips were fixed with absolute methanol at −20°C. Non-specific binding was avoided by saturating samples with 4% BSA in PBS. Coverslips were then incubated overnight at 4°C with anti-FLAG primary antibody followed by anti-FITC secondary antibody incubation for 1 hour at room temperature. Samples were analyzed with a Zeiss Axio Imager.Z1 microscope, equipped with 60x/NA 1.40 optics and Apotome apparatus, coupled to a computer driven Zeiss AxioCAM digital camera (MRm), using Zeiss Axio Vision (4.4) software. All images were taken at similar exposures within an experiment for each antibody. Images were processed using Adobe Photoshop 7 (Adobe Systems).

### Multiple cytokine assay

Culture media derived from untransfected or transfected U2-OS were collected 24 or and 72 hours after transfection and proteins were measured by the Bio-Plex Pro Human 27-plex Assay kit (Bio-Rad Laboratories), and by the TGF-beta 3-plex kit (Bio-Rad Laboratories) following manufacturer's instructions. The TGF-beta kit was further used to test serum levels of TGFbeta 1, 2 and 3 in four unrelated MADA patients, carrying the R527H LMNA mutation, and in 22 healthy blood donors, included as controls.

### Osteoclastogenesis

Osteoclasts were obtained from peripheral blood monocytes (PBMC) as described previously [11]. Fresh buffy coats (AVIS, Bologna, Italy) were diluted with PBS, layered over Hystopaque (Sigma, St. Louis, MO), and centrifuged at 900g for 30 minutes. The mononuclear cells were extracted from the interphase of the PBS and Hystopaque and centrifuged at 400g for 5 minutes. Cells were rinsed in PBS and seeded on tissue-culture glass or plastic ware in D-MEM supplemented with 10% FCS and incubated at 37°C in a humidified 5% CO_2_ atmosphere. Cells were seeded at the density of 3,000,000/cm^2^. After 1 hour, medium was discarded and replaced with conditioned medium from confluent cultures of WT- or R527H-transfected U2-OS cells (25% conditioned medium and 75% normal medium). Conditioned medium was replaced every 4 days for 14 days. In order to verify the differentiation of mononuclear cells to osteoclasts, after 8 days of culture, cells were analyzed for tartrate resistant acid phosphatase (TRACP) activity by cytochemistry (Acid Phosphatase Leukocyte assay, Sigma), and stained with Hoechst 33258 (1.25 g/ml). TRACP-positive cells containing 3 or more nuclei were considered as differentiated osteoclasts.

### Statistical evaluation

The data are presented as mean values from three separate experiments +/– standard deviation. Data were statistically analyzed by a Dunnet test after one-way analysis of variance (ANOVA) at a level of significance of *p* < 0.05 vs. control samples.
